# Continuous theta burst stimulation over right cerebellum for speech impairment in Parkinson’s disease: study protocol for a randomized, sham-controlled, clinical trial

**DOI:** 10.3389/fnagi.2023.1215330

**Published:** 2023-08-15

**Authors:** Xiaoxia Zhu, Guangyan Dai, Meng Wang, Mingdan Tan, Yongxue Li, Zhiqin Xu, Di Lei, Ling Chen, Xi Chen, Hanjun Liu

**Affiliations:** ^1^Department of Rehabilitation Medicine, The First Affiliated Hospital, Sun Yat-sen University, Guangzhou, China; ^2^Department of Radiology, The First Affiliated Hospital, Sun Yat-sen University, Guangzhou, China; ^3^Department of Neurology, The First Affiliated Hospital, Sun Yat-sen University, Guangzhou, China; ^4^Guangdong Provincial Key Laboratory of Brain Function and Disease, Zhongshan School of Medicine, Sun Yat-sen University, Guangzhou, China

**Keywords:** Parkinson’s disease, hypokinetic dysarthria, cerebellum, continuous theta burst stimulation, auditory-vocal integration

## Abstract

**Background:**

Speech impairment is a common symptom of Parkinson’s disease (PD) that worsens with disease progression and affects communication and quality of life. Current pharmacological and surgical treatments for PD have inconsistent effects on speech impairment. The cerebellum is an essential part of sensorimotor network that regulates speech production and becomes dysfunctional in PD. Continuous theta-burst stimulation (cTBS) is a non-invasive brain stimulation technique that can modulate the cerebellum and its connections with other brain regions.

**Objective:**

To investigate whether cTBS over the right cerebellum coupled with speech-language therapy (SLT) can improve speech impairment in PD.

**Methods:**

In this randomized controlled trial (RCT), 40 patients with PD will be recruited and assigned to either an experimental group (EG) or a control group (CG). Both groups will receive 10 sessions of standard SLT. The EG will receive real cTBS over the right cerebellum, while the CG will receive sham stimulation. Blinded assessors will evaluate the treatment outcome at three time points: pre-intervention, post-intervention, and at a 12-week follow-up. The primary outcome measures are voice/speech quality and neurobehavioral parameters of auditory-vocal integration. The secondary outcome measures are cognitive function, quality of life, and functional connectivity determined by resting-state functional magnetic resonance imaging (fMRI).

**Significance:**

This trial will provide evidence for the efficacy and safety of cerebellar cTBS for the treatment of speech impairment in PD and shed light on the neural mechanism of this intervention. It will also have implications for other speech impairment attributed to cerebellar dysfunctions.

**Clinical trial registration:**

www.chictr.org.cn, identifier ChiCTR2100050543.

## Introduction

Parkinson’s disease (PD) is a neurodegenerative disease that affects motor and non-motor functions, including speech. Nearly 90% of patients with PD are affected by motor speech disorders (Logemann et al., 1978; Ho et al., 1998), characterized by hypokinetic dysarthria that involves reduced voice loudness and pitch, speech dysfluency, imprecise articulation, and monotone (Logemann et al., 1978). Motor speech disorders can occur early in the disease progression (Stewart et al., 1995) or even during the prodromal stages of PD (Postuma et al., 2012; Rusz et al., 2021), and their severity tends to decline progressively (Holmes et al., 2000; Skodda et al., 2009, 2011). These disorders lead to impaired communication function and reduced quality of life (Miller et al., 2006; Pell et al., 2006; Hall et al., 2011).

Despite the prevalence of motor speech disorders in PD, the current treatment options are limited and inconsistent. Dopaminergic medication, the main pharmacological therapy for motor symptoms in PD, has shown mixed effects on speech function, with some studies reporting slight improvement in acoustic-phonatory characteristics, respiratory parameters, and speech intelligibility (Goberman et al., 2002; De Letter et al., 2007; Ho et al., 2008; Okada et al., 2015), whereas others showing no effect or even a worsening of speech function (Plowman-Prine et al., 2009; Fabbri et al., 2017). These findings suggest that other underlying mechanisms, beyond dopaminergic deficits, may contribute to speech impairment in PD. Deep brain stimulation (DBS) of the subthalamic nucleus (STN), a surgical therapy for motor symptoms in advanced PD, has also shown variable effects on speech function, with some studies reporting improvement in oral motor and voice features (Gentil et al., 2003; Dromey et al., 2010; Sidtis and Sidtis, 2017) whereas others reporting no effect or even a deterioration of speech function (Tripoliti et al., 2011; Xu et al., 2018; Tanaka et al., 2021; Vos et al., 2021; Catalano Chiuve et al., 2022). Moreover, there is evidence that the effects of DBS on speech function may depend on the stimulation parameters or task demands (Klostermann et al., 2008; Tripoliti et al., 2008). This discrepancy in response to DBS between limb motor symptoms and speech impairment further underscores the notion that speech impairment in PD may involve mechanisms that are not strictly dopaminergic in nature. As such, it is important to consider non-pharmacological or non-invasive treatment approaches for managing speech impairment in PD.

To date, speech-language therapies (SLTs) are often recommended as a complementary intervention. One of SLTs specifically designed for hypokinetic dysarthria in PD is the Lee Silverman Voice Treatment (LSVT^®^ LOUD) (Ramig et al., 1996), which aims to enhance vocal loudness and improve speech quality. Previous studies have demonstrated immediate and long-term (12–24 months) benefits of LSVT^®^ LOUD in terms of vocal loudness, pitch variability, and speech intelligibility (Ramig et al., 2001; Sapir et al., 2007; Li et al., 2021). However, this treatment is constrained by high-effort and intensive vocal training, which can lead to a high dropout rate among patients. Therefore, there is a need to develop new alternative methods that provide more accessible and sustainable benefits for speech impairment in PD.

Repetitive transcranial magnetic stimulation (rTMS) is a non-invasive method that can modulate cortical excitability by applying magnetic pulses over the scalp. Several meta-analyses have shown a significant medium-sized effect of rTMS on alleviating PD motor symptoms (Chou et al., 2015; Chung and Mak, 2016). However, there is limited and conflicting evidence for rTMS on speech impairment in PD. Early studies focused on applying high-frequency rTMS over certain brain regions in patients with PD, such as the left primary orofacial sensorimotor (SM1), primary motor area (M1) associated with the hand or mouth, and the left dorsolateral prefrontal cortex (DLPFC), but found inconsistent effects on their voice quality and loudness (Dias et al., 2006; Hartelius et al., 2010; Eliasova et al., 2013). For example, while improved vocal pitch and loudness, tongue movement, and voice quality in patients with PD were found after high-frequency rTMS over the left SM1 or the M1-mouth area (Dias et al., 2006; Eliasova et al., 2013), no such benefits were not found when high-frequency rTMS was applied over the left DLPFC or the M1-hand area (Dias et al., 2006; Hartelius et al., 2010; Eliasova et al., 2013). Recently, low-frequency rTMS over the right superior temporal gyrus (STG) led to improved speech articulation in patients with PD, which was associated with enhanced right STG activation during sentence reading (Brabenec et al., 2019) and resting-state functional connectivity (FC) between STG and SM1 (Brabenec et al., 2021). Therefore, there is mixed evidence regarding the efficacy of TMS intervention for PD speech impairment, which may be related to variations in stimulation protocol and treatment outcomes. The choice of stimulation parameters, including modality and location, may have differential impacts on the intervention effects on speech impairment in PD. On the other hand, conventional outcome measures, such as acoustic [e.g., fundamental frequency(*f*_o_), intensity, formant frequency] and/or perceptual (e.g., phonetics score, speech intelligibility) assessments (Dias et al., 2006; Eliasova et al., 2013; Brabenec et al., 2021), may not fully capture the multifaceted aspects of speech improvement and the dynamic nature of speech communication. Therefore, future studies should carefully consider these factors when designing and evaluating rTMS interventions for speech disorders in PD.

Speech production is a complex process that requires the coordination of both cortical and subcortical regions (Hickok, 2012). Within this network, the cerebellum plays an important role in supporting various aspects of speech production, such as motor planning, timing, sequencing, coordination and error correction (Ackermann et al., 1998; Chen and Desmond, 2005; Ben-Yehudah et al., 2007; Manto et al., 2012; Marien et al., 2014). Neuroimaging studies have revealed altered cerebellar activity and abnormal functional connectivity between cerebellum and other brain regions in PD (Yu et al., 2007; New et al., 2015; Yang et al., 2016; Manes et al., 2018). On the other hand, the cerebellum has been shown to be activated during sensorimotor control of speech production, as evidenced by increased cerebellar activity in response to first formant (*F*_1_) perturbations (Tourville et al., 2008) and jaw perturbations (Golfinopoulos et al., 2011) during speech production. Clinically, patients with spinocerebellar ataxia (SCA) have shown reduced adaptive responses to speech *F*_1_ perturbations (Parrell et al., 2017) and enhanced reflexive responses to vocal pitch perturbations (Houde et al., 2019; Li et al., 2019). Similarly, impaired sensorimotor control of vocal production has been found in patients with PD, as reflected by abnormal vocal compensations for altered loudness, pitch, or formant during vocal/speech production (Liu et al., 2012; Chen et al., 2013; Huang et al., 2016; Mollaei et al., 2016). Together, these findings suggest that the cerebellum may be impaired or dysfunctional in patients with PD, which may contribute to their speech impairment.

Given the importance of the cerebellum in speech production and the evidence of cerebellar dysfunction in PD, it is reasonable to hypothesize that modulating cerebellar activity could improve speech impairment in patients with PD. To test this hypothesis, the present protocol will apply continuous theta burst stimulation (cTBS), a specific form of rTMS that induces long-lasting inhibitory effects on neuronal excitability (Huang et al., 2005), over the right cerebellum in patients with PD and evaluate their speech function before and after stimulation. The present protocol chooses the cTBS protocol based on the observation of enhanced auditory-motor integration for vocal pitch regulation in patients with SCA (Lin et al., 2022) as a consequence of inhibiting cerebellar activity. The right cerebellum is selected as the target site based on the following considerations. Activation of the right cerebellum has been observed during verbal generation tasks (Riecker et al., 2000; Stoodley, 2012), verbal working memory (Riva and Giorgi, 2000), voiced speech (Schulz et al., 2005), and compensatory adjustment of speech *F*_1_ (Tourville et al., 2008). Moreover, studies employing cTBS have reported impaired verbal working memory and reduced accuracy in lexical tasks when the right cerebellum was stimulated, a phenomenon that was not observed when cTBS was applied over the contralateral region (Argyropoulos, 2011; Tomlinson et al., 2014). Additionally, clinical evidence has shown that damage to the right cerebellum is often associated with impaired speech articulation and planning (Silveri et al., 1998; Ackermann et al., 2007). More importantly, recent tDCS and cTBS studies have established a causal relationship between the right cerebellum and speech production. For example, anodal tDCS and cTBS over the right cerebellum resulted in increased (Peng et al., 2021) and decreased (Lin et al., 2022) vocal compensations for pitch perturbations, respectively. Interestingly, this causal relationship was absent when the left cerebellum was stimulated with TBS (Liu et al., 2022). In addition, increased speech compensations for *F*_1_ perturbations were found when anodal tDCS was applied over the right cerebellum (Lametti et al., 2018).

In this protocol, patients with PD will be divided to two groups: one group will receive real cTBS over the right cerebellum, while the other group will receive sham stimulation. Before and after TMS intervention, they will perform a battery of speech tasks to assess their voice loudness and pitch, speech fluency, articulation accuracy, and prosodic variation. We will also evaluate auditory-motor control of vocal production using the frequency-altered feedback (FAF) paradigm (Burnett et al., 1998), which involves manipulating auditory feedback during vocalization and measuring compensatory changes in vocal output. Recent evidence has shown a significant correlation between reduced compensatory responses to vocal pitch errors and improved vocal intensity during passage reading in patients with PD following LSVT^®^ LOUD (Li et al., 2021). To investigate the neural mechanisms underlying the effects of cerebellar cTBS on speech impairment in PD, we will employ resting-state functional magnetic resonance imaging (rs-fMRI) to measure FC between the cerebellum and other brain regions. This protocol will provide new insights into the cerebellar contribution to PD speech disorders and potential therapeutic interventions.

## Materials and methods

### Study design

This study design adheres to a randomized, sham-controlled, single-center clinical trial, in accordance with the Standard Protocol Items Recommendations for Intervention Trails (SPIRIT) guideline (Chan et al., 2015). The study will be conducted in the Department of Rehabilitation Medicine at The First Affiliated Hospital of Sun Yat-sen University, China. The research protocol was approved by the Ethics Committee of FAH-SYSU (No. [2020]471). The trial was prospectively registered on www.chictr.org.cn under the registration number ChiCTR2100050543.

**FIGURE 1:**
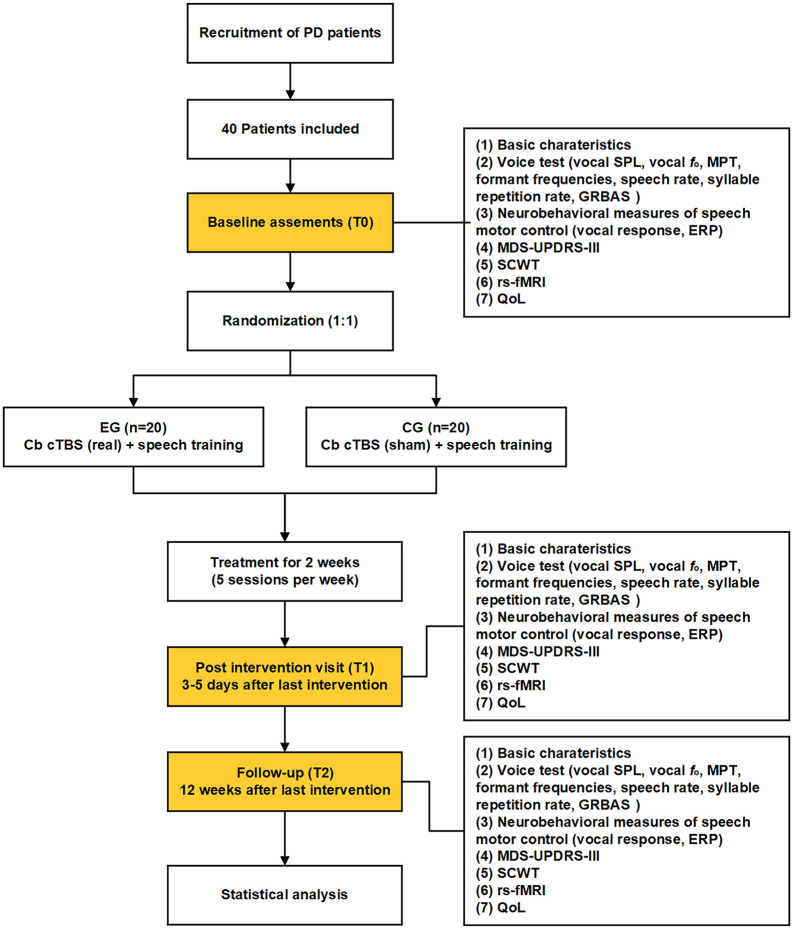
Flow chart of the present study protocol. EG, experimental group; CG, control group; Cb, cerebellum; cTBS, continuous theta burst stimulation; SPL, sound pressure level; MPT, maximum phonation time; ERP, event-related potential; rs-fMRI, resting-state functional magnetic resonance imaging; GRBAS, grade, rough, breathiness, asthenia and strain; SCWT, Stroop Color and Word Test; QoL, quality of life.

Patients will be randomly assigned to one of two groups: an experimental group (EG) and a control group (CG). While all participants will receive routine SLT, the EG will also receive real TMS while the CG will receive sham TMS. The intervention consists of 10 sessions over 2 weeks. The outcomes will be measured at three time points: baseline (T0), 3–5 days before the first session; post-intervention (T1), 3–5 days after the last session; and follow-up (T2), 12 weeks after the last session. The flow chart of this study protocol is shown in Figure 1.

### Participants

#### Inclusion criteria

We will recruit patients who meet the following criteria: (a) diagnosed with idiopathic PD by a neurologist according to the UK Parkinson’s disease Society Brain Bank (Hughes et al., 1992); (b) aged 50–80 years; (c) right-handedness; (d) Mandarin as their primary language; (e) normal hearing function; (f) Modified Hoehn & Yahr (mH&Y) scale of 1.5–3; (g) Mini-Mental State Examination (MMSE) score ≥ 26; (h) no previous speech therapy; and (i) stable dopaminergic medication assessed by Levodopa Equivalent Daily Dose (LEDD) for a minimum of 4 weeks preceding baseline assessment.

#### Exclusion criteria

We will exclude patients who have any of the following conditions: (a) contraindications for MRI and TMS (e.g., claustrophobia, cardiac pacemaker); (b) focal neurological disorders (e.g., epilepsy, stroke, brain injury or tumor) or related psychiatric disorders; (c) history of brain surgery such as deep brain stimulation; or (d) unstable medical conditions.

#### Sample size

To date, none of studies has investigated the effects of cerebellar cTBS on speech impairment in patients with PD. However, our previous study reported positive effects of cerebellar cTBS on impaired auditory-vocal integration in patients with SCA (Lin et al., 2022), who similarly showed abnormally enhanced vocal compensations for pitch perturbations (Li et al., 2019). Based on the obtained partial η^2^ value of 0.559 in that study, we performed an estimation of effect size using G*Power software (version 3.1). Specifically, we used *a priori* F-tests for repeated-measures within-between interaction to determine the required sample size for our study. Our calculations indicated that a total sample size of 16 participants would be necessary to achieve a power of 0.95 and an alpha level of 0.05, considering the presence of two groups. The chosen sample size allows us to adequately investigate the research question while considering resource constraints. To account for potential dropouts and data quality issues, we increased the sample size by 20%, resulting in a final sample size of at least 10 participants per group. However, it is important to note that this sample size calculation is based on the immediate aftereffects of cerebellar cTBS on speech impairments in patients with SCA. Therefore, this study protocol will recruit a total of 40 participants, with 20 participants allocated to each group, for a more conservative approach.

### Randomization, allocation, and blinding

We will use block randomization with a block size of 4 and a 1:1 allocation ratio to assign patients with PD who pass the screening to either the experimental or control group. Randomization will be stratified by gender and age. An independent statistician, who is not be involved in participant enrollment, assessment, or intervention, will generate the randomization sequence. Allocation concealment will be ensured using sealed opaque envelopes, and each envelope will bear a serial number on the outside and a group number on the inside. After baseline assessments, an independent research assistant will open the envelopes in order and assign participants to one of the two groups based on the group number enclosed. Participants, outcome assessors, and data analysts will be blinded to the allocation and intervention.

### Intervention

All participants will receive routine SLT and 10 sessions of TMS intervention (real or sham cerebellar cTBS) over 2 weeks. The SLT will consist of 30 min of group speech therapy delivered by speech-language pathologists (SLPs), with four patients per group. The SLT will aim to improve voice/speech quality through exercises involving breathing control, vowel vocalization, reading, and singing. Notably, the SLPs will personalize the training materials based on the family background and interests of patients with PD and adjust the program during training according to individual conditions. The administration of cerebellar cTBS will precede the SLT. All interventions will be conducted at the same time of day when the patients are in their “ON” state, and their medication will be kept constant throughout the trial.

### Transcranial magnetic stimulation

Cerebellar cTBS will be administered using a magnetic stimulator (YIRUIDE CCY-1, Wuhan, China) with a 70 mm figure-of-8 coil. Neuronavigation software (Visor 2.0, ANT Neuro, Netherlands) in conjunction with a motion tracking system (Polaris Spectra, NDI, Canada) to ensure accurate and consistent coil positioning over the target areas. Before stimulation, we will measure the active motor threshold (AMT) from the right first dorsal interosseous muscle (FDI) by applying single-pulse TMS over the left M1 for each participant. The AMT is defined as the minimum stimulation intensity required to elicit motor-evoked potentials (MEPs) > 200 μv in tonically contracted muscles (10% of maximum contraction) in at least 5 out of 10 consecutive trials. A standard cTBS protocol that consists of 600 pulses in a theta burst pattern (bursts of 3 pulses at 50 Hz repeated every 200 ms) (Huang et al., 2005) will be delivered at 80% of AMT.

The VII lobe of the right cerebellum will be targeted as a stimulation site, as previous research has shown improved auditory-vocal integration in patients with SCA when cTBS was applied over this region (Lin et al., 2022). The mean Montreal Neurological Institute (MNI) coordinates are (42, -56, -30) (Stoodley, 2012). Structural MRI data from each participant will be imported into the neuronavigation software and segmented to obtain a realistic head model of the scalp and brain. During the navigation, the coil will be placed tangentially to the scalp and adjusted to reach the target markers. The neuronavigation software will provide distance and angle values as figures of merit for targeting. When the distance and angle between the coil and target are below predefined thresholds (distance < 2 mm, angle < 10°), the values will turn green to indicate the desired targeting precision. For the sham group, the same experimental parameters will be used except that the coil will be tilted at 90° with only the edge touching the scalp. This sham procedure ensures similar noise and scalp sensation but does not induce cortical activation.

### Outcome assessment

A comprehensive range of behavioral, neurophysiological, and neuroimaging measures will be used to investigate the effects of cerebellar cTBS on speech functions in patients with PD. In addition, other measures such as cognitive function and quality of life will also be assessed. All outcome measures will be collected at pre-intervention, post-intervention and follow-up assessments, with data acquired in the “ON” medication state at each time point. To ensure that patients with PD remain in the ON medication state throughout the experiment, clear instructions will be provided to them to adhere to their regular medication schedule for the duration of the study. And we will prioritize assessments involving voice/speech function and the FAF paradigm, followed by the assessment of cognitive function and questionnaires. These evaluations will take between 60 to 90 min, a timeframe that fits within the effective period of levodopa. Table 1 provides an overview of all measures and their corresponding time points.

**TABLE 1:** Schedule of recruitment, intervention and assessment.

	Study period				
	Enrollment/Baseline	Allocation	Post-allocation		
Timepoint	W-1	0	W1	W2	W14
**Enrolment**					
Eligibility screen	X				
Informed consent	X				
Randomization	X				
Allocation		X			
**Intervention**					
Real cTBS + rehabilitation			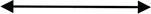		
Sham cTBS + rehabilitation			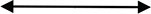		
**Assessments**					
**Basic characteristics**					
Demographic characteristics	X				
Medical history	X			X	X
Modified H&Y stage	X			X	X
MMSE	X			X	X
Hearing test	X			X	X
**Primary outcomes**					
Voice quality	X			X	X
Vocal response	X			X	X
ERP	X			X	X
**Secondary outcomes**					
MDS-UPDRS-III	X			X	X
SCWT	X			X	X
QoL	X			X	X
fMRI scanning	X			X	X
**Safety**			X	X	X

### Primary outcomes

#### Voice quality

Participants will perform an array of speech tasks designed to assess various aspects of their speech functions. These tasks consist of sustained vowel phonation, reading a specified passage (Chinese version of “The North Wind and the Sun” that was developed by the International Phonetic Association), spontaneous speech production, a pitch range task that involves gliding the voice from the lowest to the highest pitch and vice versa, as well as a diadochokinetic task that requires rapid and alternative articulatory movements (“pa-tak-ka”). Vocal sound pressure level (SPL), voice *f*_o_, maximum phonation time (MPT), and formant frequencies will be analyzed for sustained vowel phonation. Vocal SPL, speech rate, and syllable repetition rate will be analyzed for speech tasks.

In addition, perceptual voice quality will be assessed using the Japanese GRBAS scale that consists of 5 items: grade, rough, breathiness, asthenia and strain (Moro-Velazquez et al., 2021). To minimize inter-rater variability and enhance reliability of this perceptual evaluation, we will involve three experienced raters, each skilled in evaluating speech disorders associated with PD, to independently assess the speech samples using the GRBAS scale. These raters independently evaluate all speech samples, and their scores are averaged to obtain the final GRBAS ratings. All raters will undergo an alignment process to standardize scoring methods and criteria prior to the start of the study. Furthermore, intra-class correlation coefficients (ICCs) will be calculated to quantitatively measure inter-rater reliability and control for any potential variability.

#### Neurobehavioral changes of vocal motor control

The FAF paradigm will be used to measure neurobehavioral changes in auditory-vocal integration in patients with PD before and after TMS intervention, which will reflect their ability to integrate auditory feedback and motor control for speech processing. They will be instructed to produce sustained phonations while exposed to unexpected pitch perturbations in auditory feedback, with vocal and electroencephalographic (EEG) signals recorded simultaneously (see details below). The magnitude and latency of vocal responses will be measured as behavioral responses, while the P1, N1, and P2 components of the event-related potentials (ERPs) will be measured as neurophysiological responses.

### Secondary outcomes

#### Cognitive function

As a secondary outcome, cognitive function will be assessed using a neuropsychological test that measures attentional control and executive functions. It is noteworthy that emerging research over recent years has progressively expanded our understanding of cerebellar function from motor control to cognitive processes, including but not limited to attentional control (Esterman et al., 2017), working memory (Marvel and Desmond, 2010), and affective regulation (Schmahmann and Sherman, 1998). Specifically, auditory-motor integration for speech production has been shown to bear significant associations with attentional control (Liu et al., 2015), working memory (Guo et al., 2017), and executive function (Ranasinghe et al., 2017). This relationship is further supported by recent studies that provide causal evidence supporting the notion that a top-down neural mechanism mediated by the prefrontal cortex exerts an inhibitory control over auditory-motor processing of speech production (Liu et al., 2020; Chang et al., 2023). It is thus plausible that cTBS over the right cerebellum may yield improvements in both speech production and cognitive function in patients with PD, which will provide insights into the contributions of neurocognitive functions to speech production. To address this point, the Stroop Color and Word Test (SCWT) will be used to assess the ability of attentional control and executive function (Hsieh et al., 2008), during which participants will be asked to name the ink colors of color words printed in discordant ink. The Stroop effect will be calculated as the delay in reaction time between automatic and controlled processing of information.

#### Quality of life

The Parkinson’s Disease Questionnaire (PDQ-39) will be used as a secondary outcome to evaluate the impact of PD on quality of life. It assesses the frequency of difficulties experienced by patients with PD across 8 dimensions of daily living, including relationships, social situations, communication, functioning, and wellbeing. In addition, the Voice Handicap Index (VHI) will be used to evaluate voice-related quality of life.

### Resting-state fMRI

To gain further insight into the neural mechanisms underlying the effect of cerebellar cTBS on speech impairment in patients with PD, rs-fMRI will be used to investigate differences in neural activity patterns between the two groups. Rs-fMRI has emerged as a valuable tool for monitoring disease progression and treatment outcomes in PD (Baggio et al., 2015). In this protocol, rs-fMRI will be used to detect and assess changes in FC, which reflects the local synchronization of spontaneous neural activity, in patients with PD following cerebellar cTBS interventions.

### Data collection and processing

#### Demographic data collection

Demographic information, including age, gender, educational level, hearing function, disease stage, cognitive function, medical history, medication list, and comorbidities, will be collected from all participants. This information will be documented in paper forms and subsequently transferred to electronic files. Study assessors will receive thorough training before conducting the assessments to ensure accurate and consistent data collection.

#### Acoustic data acquisition

All patients with PD will perform speech tasks in a sound-attenuated room under following conditions: sustained vowel phonation, reading a specified passage, spontaneous speech production, a pitch range task, and a diadochokinetic task. Voice signals will be collected via a dynamic microphone (DM2200, Takstar Inc., Huizhou, China) at 44 kHz and transmitted to a computer using Praat software (Boersma, 2001). Acoustic parameters, including vocal SPL, voice *f*_o_, MPT, formant frequencies, speech rate, and syllable repetition rate (Ramig et al., 1996; Wang et al., 2006), will be extracted and analyzed using Praat for statistical analyses.

### Data collection, preprocessing, and analysis

#### Vocal response data

The FAF-based vocal production task will be conducted in a sound-attenuated room. Patients with PD will be instructed to produce sustained phonations of the vowel/u/for approximately 3–4 s at their comfortable pitch and loudness level. During each vocalization, they will hear their voice pitch unexpectedly shifted upward or downward twice by 200 cents (100 cents = 1 semitone), with each perturbation lasting for 200 ms. The first pitch perturbation will occur 900–1,200 ms after the vocal onset, followed by the second pitch perturbation occurring 1,200–1,500 ms after the first one. All patients with PD will take a pause of 3–4 s between successive vocalizations to avoid vocal fatigue. They will produce 100 consecutive vocalizations, resulting in a total of 100 trials for +200 cents and −200 cents pitch perturbations.

To partially mask airborne and bone-conducted feedback, the recording system will be acoustically calibrated so that participants hear voice feedback with a gain of 10 dB SPL higher than their vocal output (Behroozmand et al., 2009; Liu et al., 2011). During the experiment, voice signals will be transduced by a dynamic microphone (DM2200, Takstar Inc., Huizhou, China), amplified with a MOTU Ultralite Mk3 Firewire audio interface (Cambridge, MA, USA), and pitch-shifted through an Eventide Eclipse Harmonizer (Little Ferry, NJ, USA). A custom-developed MIDI software program (Max/MSP v.6.1 by Cycling 74, San Francisco, CA, USA) will control the Eventide Eclipse Harmonizer to shift voice pitch and generate transistor-transistor logic (TTL) control pulses to mark the onset of the pitch perturbation. The pitch-shifted voice signals will be further amplified by an ICON Neo Amp headphone amplifier (Middleton, WI, USA) and delivered to participants through insert earphones (ER-1, Etymotic Research Inc., Elk Grove Village, IL, USA). The original and pitch-shifted voice signals, as well as TTL pulses, will be recorded at 10 kHz by a PowerLab A/D converter (model ML880, AD Instruments, Castle Hill, NSW, Australia) using LabChart software (v.7.0, AD Instruments).

The magnitude and latency of vocal responses to pitch perturbations will be measured using a custom-developed IGO RPRO software program (v.6.0 by WaveMetrics Inc., Lake Oswego, OR, USA) (Liu et al., 2012; Li et al., 2021). Voice *f*_o_ contours in hertz will be extracted from voice signals using Praat software and converted to the cent scale using the formula: cents = 100 × [12 × log2(*f*_o_/reference)] [reference = 195.997 Hz (G3)]. Voice *f*_o_ contours will be segmented into epochs ranging from 200 ms before to 700 ms after the onset of the pitch perturbation and visually inspected to identify and reject any artifacts. Individual trials contaminated by unexpected vocal interruptions or signal processing errors will be regarded as bad trials and excluded from subsequent analyses. Trials without artifacts will be averaged to generate an overall response for each condition. The magnitude and latency of a vocal response will be, respectively measured as the maximum or minimum value in cents and the corresponding time in milliseconds when the voice *f*_o_ contour reaches its peak value.

#### EEG data

EEG data will be recorded using a 64-electrode Geodesic Sensor Net (Electrical Geodesics Inc., Eugene, OR, USA) connected to a high input-impedance Net Amps 400 amplifier (Electrical Geodesics Inc., Eugene, OR, USA). The EEG signals will be referenced to the vertex (Cz) and digitally sampled at 1 kHz using NetStation software (v.5.4, Electrical Geodesics Inc., Eugene, OR, USA). TTL pulses will be sent to the EEG recording system via an experimental synch Deutsches Institut für Normung (DIN) for synchronization of voice and EEG signals. The impedance levels of individual sensors will be kept below 50 kΩ throughout the recording, as this amplifier accepts scalp-electrode impedances up to 60 kΩ (Ferree et al., 2001).

The EEG signals will be analyzed offline using NetStation software. They will be band-pass filtered with cut-off frequencies of 1–20 Hz and segmented into epochs ranging from 200 ms before to 500 ms after perturbation onset. An artifact detection procedure will be applied to identify bad trials, during which trials whose voltage values exceed ± 55 μv of the moving average over an 80-ms window will be rejected from further analysis. After re-referencing to the average of the electrodes on each mastoid, artifact-free trials will be averaged and baseline-corrected (−200 ms to 0) to generate an overall ERP response for each condition. A total of 24 electrodes in three regions of interest (ROIs) will be selected for statistical analysis (Dai et al., 2022; Lin et al., 2022): frontal area, including AF3, AFz, AF4, F5, F3, F1, Fz, F2, F4, and F6; fronto-central area, including FC5, FC3, FC1, FCz, FC2, FC4, and FC6; and central area, including C5, C3, C1, Cz, C2, C4, and C6. The amplitudes and latencies of the P1, N1 and P2 components will be extracted from the averaged ERPs for each ROI.

#### MRI data

MRI data will be acquired using a 3.0 T Siemens magnetic resonance system (SIEMENS MAGNETOM Prisma, Germany). Participants will be instructed to stay awake with their eyes closed, refrain from thinking of anything, and remain motionless during the scanning session. Memory foam padding and earplugs will be provided to minimize head motion and scanner noise. To minimize fatigue and ensure that patients are in the “ON” state during MRI scanning, the scanning session will be scheduled on a different day from the other assessments. The following sequences will be acquired: (1) a high-resolution structural T1-weighted MPRAGE sequence will be used to acquire anatomical images with the following parameters: repetition time (TR) = 2,000 ms; echo time (TE) = 1.76 ms; flip angle = 8°; field of view (FOV) = 260 × 260 mm^2^; slice thickness = 0.6 mm; number of slices = 224; voxel size = 0.8 × 0.8 × 0.8 mm^3^. (2) resting-state fMRI data will be acquired using an EPI imaging sequence with the following parameters: TR = 2,000 ms; TE = 30 ms; flip angle = 90°; FOV = 224 × 224 mm^2^; voxel size = 3.5 × 3.5 × 3.5 mm^3^.

Statistical Parametric Mapping (v.12.0, Wellcome Trust Centre for Neuroimaging, London, UK) will be used for the preprocessing of rs-fMRI data on the MATLAB platform (MathWorks Inc., Natick, MA, USA). The preprocessing steps will include head motion correction, slice timing correction, spatial normalization, and smoothing. After preprocessing, the REST software (Song et al., 2011) will be used for connectivity analysis of brain functions. Regional homogeneity (ReHo) will be calculated using the Kendall coefficient of concordance (KCC) between the time series of a given voxel and those of its 26 neighboring voxels to reflect the local synchronization of spontaneous brain activity (Zang et al., 2004). ReHo maps between groups will be compared using voxel-wise two-sample *t*-tests in SPM with a significance level of *p* < 0.05. FC, which reflects the temporal correlation of spontaneous brain activity between different regions, will be calculated by Pearson’s correlation coefficient between the time series of a ROI and those of all other voxels in the brain. The right cerebellar hemisphere will be used as the ROI because it has been linked to speech and language functions (Marien et al., 2014; Moberget et al., 2014; Lesage et al., 2017; Argyropoulos et al., 2019; Diedrichsen et al., 2019). The ROI will be centered at MNI coordinate (42, -56, -30) and defined by a sphere with a radius of 6 mm. Correlation coefficients will be transformed to z-scores using Fisher’s r-to-z transformation to obtain z-FC maps for each participant. The contrast between z-FC maps post- and pre-intervention for each participant will be calculated to obtain Δz-FC maps, which will be used for a two-sample *t*-test to compare FC changes between the EG and CG groups. The statistical criterion will be set at *p* < 0.05, and false discovery rate (FDR) will be used for multiple comparisons correction.

### Statistical analysis

Statistical analyses of clinical and behavioral parameters will be performed using SPSS software (v. 20.0). All patients with PD who have at least one post-intervention assessment will be included in the analysis. Gender differences between groups will be assessed using a chi-square test. Baseline measures will be compared between groups using two-sample *t*-tests. A 2 × 3 two-way repeated-measures analysis of variance (RM-ANOVA) with factors of group (real cTBS, sham cTBS) and time (pre-intervention, post-intervention, 12-week follow-up) will be used to investigate the effects of cerebellar cTBS on speech functions, neurobehavioral changes of vocal motor control, and quality of life. *Post hoc* pairwise correction will be performed using Bonferroni comparison adjustment. Pearson analysis will be used to examine correlations between neurophysiological and behavioral measures. A *p*-value < 0.05 in a two-tailed test will be considered statistically significant.

### Data management and monitoring

Data management and monitoring procedures will be implemented to ensure the integrity and security of the collected data. Questionnaires and scales data will be digitalized and stored in a secure file. A second research staff member will verify the accuracy of data entry to avoid errors. EEG, MRI, and other digital data, including output files from computer-based tasks, will be stored on password-protected research computers. Paper-based questionnaires and forms for each participant will be securely stored and sorted by participant ID number for easy access at each stage of the study. Forms containing personal names and data will be stored separately in a locked cabinet. Files containing personal data will be encrypted and accessible only to authorized staff involved in the project. MRI data will undergo pseudonymization to protect participant privacy before analysis.

### Safety

Any adverse events (AEs) that occur throughout the study, whether related to cerebellar c-TBS, MRI scanning, or the rehabilitation program, will be monitored and documented. AEs are defined as any undesirable medical experiences that participants may encounter during the course of the study. Transient head or scalp discomfort and facial twitching in the area of stimulation are the most common AEs associated with TMS. These effects typically resolve once the stimulation is discontinued. Although rare, seizures represent a serious AE that can occur during or immediately following TMS treatment. Seizures are usually brief, lasting less than 1 min (or up to 5 min), and do not typically result in long-term medical complications. All AEs will be reported and documented on the case report form (CRF) by the investigators. Any serious adverse event (SAE) will be promptly reported to the ethics committee, which will determine whether the participant should continue with the intervention. If there is significant deterioration in the participant’s condition, the assigned intervention will be discontinued. Participants who experience harm as a result of their participation in this trial will be provided with appropriate compensation.

## Discussion

This protocol will be the first randomized, sham-controlled trial to investigate the effects and underpinning mechanisms of cTBS over the right cerebellum on speech impairment in PD. The rationale for targeting the right cerebellum with cTBS in this protocol is based on two main considerations. First, the right cerebellum has been implicated in motor aspects of speech and language functions, including articulation, prosody, fluency, and syntax (Marien et al., 2014; Moberget et al., 2014; Lesage et al., 2017; Argyropoulos et al., 2019; Diedrichsen et al., 2019). Second, recent evidence has suggested that cTBS over the right cerebellum can modulate auditory-motor integration for controlling vocal production in patients with SCA, as indicated by reduced vocal compensations for pitch perturbations in auditory feedback (Lin et al., 2022). This protocol will provide novel insights into the potential of cerebellar TMS as a non-invasive and augmentative intervention for speech impairment in PD and contribute to advancing our understanding of the neural mechanisms involved in this intervention.

One important advantage of this protocol is the application of a neuronavigation system for accurate and reliable coil positioning over the stimulation target, namely the VII lobe of the right cerebellum. The precise location and orientation of the coil affect the site and direction of the stimulation, which are critical for the validity and reproducibility of TMS studies. Neuronavigation techniques allows for the precise, consistent, and adjustable targeting of specific brain regions based on individual structural MRI data, thereby enhancing the efficacy, reliability, and safety of TMS interventions. In contrast, previous studies that reported inconsistent effects of rTMS on PD speech disorders (Brabenec et al., 2017) targeted the stimulation site according to the international 10–20 EEG system, a conventional but inaccurate method to guide TMS coil placement based on scalp landmarks. This method may compromise the effectiveness of TMS intervention because it does not account for the anatomical variability of the human brain across individuals, which may influence cortical excitability and stimulation depth. Therefore, the use of neuronavigation techniques is recommended for precise coil positioning in TMS studies. Notably, previous studies targeting specific brain regions in PD using neuronavigation guidance have demonstrated positive outcomes, showing improved levodopa-induced dyskinesias with cTBS over the cerebellum (Koch et al., 2009) and enhanced speech articulation with low-frequency rTMS over the right STG (Brabenec et al., 2019, 2021).

In addition to acoustic and perceptual analyses of speech signals, this protocol incorporates the FAF paradigm to evaluate the effect of cerebellar cTBS on hypokinetic dysarthria in PD in terms of auditory-vocal integration. Coupled with EEG technique, the FAF paradigm measures the vocal and ERP responses to unexpected pitch changes during vocalization, which provides insights into the function of sensorimotor integration for speech processing and may offer greater sensitivity than conventional acoustic and perceptual evaluations for detecting speech impairments. Previous studies have shown that patients with PD have difficulties in integrating auditory feedback with motor systems to properly regulate their vocal production, as evidenced by significantly larger vocal adjustments for and/or greater ERP P2 responses to unexpected pitch or loudness changes relative to healthy controls (Liu et al., 2012; Chen et al., 2013; Huang et al., 2016; Mollaei et al., 2016). More importantly, recent findings have shown auditory-vocal integration in patients with PD can be modulated by SLT or non-invasive brain stimulation, as shown by normalized and reduced vocal and ERP P2 responses to pitch perturbations after LSVT^®^ LOUD (Li et al., 2021) or cTBS over the left SMA (Dai et al., 2022). Notably, patients with PD who underwent LSVT^®^ LOUD exhibited a significant correlation between reduced vocal compensations for pitch perturbations and improved vocal loudness during passage reading (Li et al., 2021). Building on these observations, this protocol hypothesizes that cTBS over the right cerebellum in patients with PD will lead to decreased vocal adjustment in response to auditory feedback perturbations, reflecting their improvement of auditory-vocal integration that enables accurate perception and appropriate correction of errors in vocal output.

This protocol also incorporates rs-fMRI to examine changes in FC between the cerebellum and other brain regions before and after TMS intervention. Rs-fMRI allows for the examination of spontaneous neural activity and functional networks without the need for task performance (Wolters et al., 2019), enabling us to investigate the neural mechanisms underlying the effect of cerebellar c-TBS on speech impairment in PD. Previous studies have shown abnormal connectivity between the cerebellum and other brain regions in patients with PD (Hacker et al., 2012; Wu and Hallett, 2013; Chen et al., 2023), which is correlated to their motor symptoms (Yoo et al., 2019) and speech impairment (New et al., 2015). Moreover, TMS studies on PD have shown significantly correlations between improvement in motor symptoms and changes in functional connectivity (Mi et al., 2020; Tinaz, 2021; Chi et al., 2023). However, few studies have used rs-fMRI to evaluate the effects of TMS on PD speech impairment. Brabenec et al. (2021) found that low-frequency rTMS over the right STG led to improved speech articulation in patients with PD, along with increased FC between the right STG and the left-sided articulatory network. These findings suggest that rTMS may modulate speech function in PD by influencing the FC of the stimulation site with regions of speech-related networks, providing valuable insights into the neural mechanisms of cerebellar c-TBS on speech impairment in PD.

However, this study also has some limitations that should be acknowledged. First, the sample size is relatively small, which may limit the generalizability and reproducibility of our findings. Second, the duration of intervention is relatively short, which may not capture the long-term effects or identify the optimal timing of cerebellar cTBS for addressing speech impairment in PD. Third, complete blinding of participants and assessors may not be possible, as some participants may experience transient side effects or sensations from real c-TBS, potentially influencing their perception of the intervention. These limitations should be addressed in future studies with larger sample sizes, longer intervention periods, and more effective blinding procedures.

## Conclusion

This protocol will provide novel insights into the role of the cerebellum in the treatment of speech impairment in PD and its potential therapeutic benefits. If our hypothesis is confirmed, this protocol will support the notion that cerebellar c-TBS can be a safe, effective, and non-invasive intervention for addressing speech impairment in PD, which can complement or enhance existing behavioral SLT approaches. This protocol will also contribute to advancing our understanding of the neural mechanisms underlying the improvement of speech impairment in PD through the modulation of the cerebello-thalamo-cortical networks using non-invasive brain stimulation techniques. The findings of this study may have important implications for the development of new therapeutic strategies and the improvement of clinical outcomes for patients with PD who suffer from speech impairments.

## Ethics statement

The studies involving humans were approved by the Ethics Committee of The First Affiliated Hospital of Sun Yat-sen University. The studies were conducted in accordance with the local legislation and institutional requirements. The participants provided their written informed consent to participate in this study.

## Author contributions

HL and XC conceived and designed the study. XZ and GD drafted the manuscript. XZ, GD, MW, MT, YL, ZX, and DL acquired the data. XC and LC helped coordinate the trial and clinical evaluation. XZ, GD, MW, and MT analyzed the data. All authors read and approved the final manuscript.
